# Genetic, psychosocial, and demographic factors associated with social disinhibition in Mexican-origin youth

**DOI:** 10.1002/brb3.236

**Published:** 2014-05-16

**Authors:** Natalie P Archer, Anna V Wilkinson, Nalini Ranjit, Jian Wang, Hua Zhao, Alan C Swann, Sanjay Shete

**Affiliations:** 1 Environmental Epidemiology and Disease Registries Section, Texas Department of State Health ServicesAustin, Texas; 2 Austin Regional Campus, University of Texas School of Public HealthAustin, Texas; 3 Department of Biostatistics, University of Texas M.D. Anderson Cancer CenterHouston, Texas; 4 Department of Epidemiology, University of Texas M.D. Anderson Cancer CenterHouston, Texas; 5 Menninger Department of Psychiatry and Behavioral Sciences, Baylor College of MedicineOne Baylor Plaza, BCM 350, Houston, Texas

**Keywords:** Genetic association study, Mexican-origin youth, sensation seeking, SNPs, social disinhibition

## Abstract

**Introduction:**

The genetic heritability for sensation-seeking tendencies ranges from 40 to 60%. Sensation-seeking behaviors typically manifest during adolescence and are associated with alcohol and cigarette experimentation in adolescents. Social disinhibition is an aspect of sensation-seeking that is closely tied to cigarette and alcohol experimentation.

**Methods:**

We examined the contribution of candidate genes to social disinhibition among 1132 Mexican origin youth in Houston, Texas, adjusting for established demographic and psychosocial risk factors. Saliva samples were obtained at baseline in 2005–06, and social disinhibition and other psychosocial data were obtained in 2008–09. Participants were genotyped for 672 functional and tagging SNPs potentially related to sensation-seeking, risk-taking, smoking, and alcohol use.

**Results:**

Six SNPs were significantly associated with social disinhibition scores, after controlling for false discovery and adjusting for population stratification and relevant demographic/psychosocial characteristics. Minor alleles for three of the SNPs (rs1998220 on *OPRM1*; rs9534511 on *HTR2A*; and rs4938056 on *HTR3B*) were associated with increased risk of social disinhibition, while minor alleles for the other three SNPs (rs1003921 on *KCNC1*; rs16116 downstream of *NPY;* and rs16870286 on *LINC00518*) exhibited a protective effect. Age, linguistic acculturation, thrill and adventure-seeking, and drug and alcohol-seeking were all significantly positively associated with increased risk of social disinhibition in a multivariable model (*P* < 0.001).

**Conclusions:**

These results add to our knowledge of genetic risk factors for social disinhibition. Additional research is needed to verify whether these SNPs are associated with social disinhibition among youth of different ethnicities and nationalities, and to elucidate whether and how these SNPs functionally contribute to social disinhibition.

## Introduction

Sensation seeking is a personality trait that is characterized by searching for novel and intense experiences and sensations, and a willingness to take risks (be they physical, financial, social, or legal) in order to obtain these experiences (Zuckerman 2007, 2009). The genetic heritability for the trait ranges from 40% to 60% (Zuckerman 2002). It is important to identify correlates of sensation-seeking tendencies from a public health perspective because they typically manifest during adolescence and are associated with health behaviors such as alcohol use, early-onset substance use, and cigarette experimentation (Martin et al. 2002; Iacono et al. 2008; Derringer et al. 2010; Wilkinson et al. 2012; Winhusen and Lewis 2013).

Social disinhibition, one aspect of sensation seeking, is a lack of social restraint that manifests itself in behaviors that disregard social conventions (Iacono et al. 2008; Weafer and Fillmore 2012), such as impolite behavior, extreme emotional outbursts, or breaking rules or laws. Because drug and alcohol use enable socially disinhibited behavior (Fillmore 2012; Weafer and Fillmore 2012), it is not surprising that this aspect of sensation seeking is tied closely to alcohol and cigarette experimentation in adolescents (McGue et al. 2006; Wilkinson et al. 2012), behaviors that have potential long-term consequences for health that are frequently initiated during adolescence and established in young adulthood (Paavola et al. 2004).

While genetic risk factors for sensation-seeking tendencies as a whole have been examined (Derringer et al. 2010; Terracciano et al. 2011), to the best of the authors' knowledge, few if any genetic studies have been conducted that focus on the specific outcome of social disinhibition. However, a couple of genetic studies have looked at genetic associations with behavioral disinhibition, which is a correlated construct that focuses more on the inability to regulate behavioral responses (McGue et al. 2013). Recently, McGue et al. 2013 completed a genome-wide association study (GWAS) examining five indicators of behavioral disinhibition: nicotine, alcohol consumption, alcohol dependence, illicit drugs, and nonsubstance-related behavioral disinhibition. This study found only one SNP (rs1868152) that attained genome-wide significance (*P* = 5 × 10^−8^), for the indicator of illicit drug use, but authors did not consider this association significant as they were analyzing five different phenotypes. Importantly, authors did find several other SNPs that could be potential candidates for future studies (McGue et al. 2013). In addition, a study by Schlaepfer et al. (2007) found a potential link between *PRKCG* and behavioral disinhibition.

In this study, we examined the contribution of over 600 single-nucleotide polymorphisms (SNPs) within candidate genes to social disinhibition among a cohort of Mexican-origin youth in Houston, Texas, adjusting for established demographic and psychosocial risk factors. It is important to understand both the genetic and nongenetic contributions to sensation-seeking behaviors among youth in order to inform the development of programs designed to prevent early smoking and alcohol use.

## Materials and Methods

### Participant recruitment

This study is based on an adolescent cohort, established between May 2005 and December 2006, of 1328 youth aged 11–13 years of age at the time of recruitment. All participants were recruited from households that form a large population-based cohort of Mexican-American households (Mano a Mano Cohort) in the Houston metropolitan area. Detailed descriptions of the Mano a Mano recruitment methodology and the establishment of the nested youth cohort have been published previously (Wilkinson et al. 2005, 2008). Briefly, a total of 3000 households with eligible youth were identified from the Mano a Mano cohort database. Of the first 1425 potential participants' parents/legal guardians contacted to inquire about participation in the study, just over 90% enrolled their children in the study (*n* = 1328 adolescents). This youth cohort is known as the Mexican-American Tobacco Use in Children study (MATCh) (Wilkinson et al. 2008). Only one eligible child per household was recruited to be part of the MATCh cohort; no siblings were selected.

### Data collection

Participants were interviewed in the home at baseline in 2005–2006, as well as 30 months later, in 2008–2009, following identical procedures. A detailed description of baseline data collection procedures has been published previously (Wilkinson et al. 2008). At baseline, youth participants provided informed assent and their parents provided informed consent. Each consenting participant completed a 5-min personal interview that collected basic demographic data (gender, age, and nativity status [US or Mexico]), and acculturation data (Norris et al. 1996). To prevent parents from overhearing their children's responses, participants completed the majority of the survey on a personal digital assistant (PDA). All participants provided buccal (saliva) samples at baseline for SNP analysis. The data on sensation seeking, most demographic indicators, and psychosocial constructs were collected during 2008–2009. Parental education data for the participants were obtained from the Mano a Mano database, and were self-reported by the parent when the parent joined Mano a Mano. All aspects of this study have been approved by the institutional review boards at the University of Texas M.D. Anderson Cancer Center and the University of Texas School of Public Health.

### Measures

The outcome variable in this study was social disinhibition, assessed by a seven-item subscale of the Sensation Seeking Scale for Children (SSSC; Russo et al. 1993). Participants endorse the choice that most describes what they like or feel, for example, “(a) I don't like being around kids who act wild and crazy” or “(b) I enjoy being around kids who sometimes act wild and crazy.” Each response option that reflects social disinhibition is worth 1 point; responses are summed to create a social disinhibition score ranging from 0 to 7 (0—least socially disinhibited, 7—most socially disinhibited). The measure has acceptable reliability (Cronbach's *α* = 0.68).

In addition to identifying SNPs that are significantly associated with social disinhibition scores among these Mexican-American youth (the exposure variables of interest), demographic and psychosocial risk factors were also examined and included as potential covariates in the model, including age, gender, parental education, level of acculturation, and two other aspects of sensation seeking—thrill and adventure seeking (TAS) and drug and alcohol seeking (DAS). We included TAS and DAS scores as covariates in the analysis because the goal of our analysis was to identify SNPs that might influence social disinhibition independently of these other aspects of sensation seeking. Because increasing age and male gender have both been associated with sensation-seeking behaviors (Wilkinson et al. 2012, 2013), age and gender were used as confounders in analyses. Parental education, rather than income, was included as a proxy for socioeconomic status (SES), as over 95% participants' parents self-reported their highest level of formal educational attainment, while fewer than 50% reported their household income. Parental educational level was analyzed as a three-level categorical variable (less than high school education, high school graduate, greater than high school education). Acculturation was assessed using four items that ascertain language used when reading, speaking at home, speaking with friends, and thinking (Norris et al. 1996). Responses are made on a 5-point scale ranging from “only Spanish” to “only English.” Each question was scored on a scale of 1–5 and averaged to produce a summary linguistic acculturation score, which ranged from 1 to 5. Higher scores indicated a greater use of English and thus a higher level of acculturation.

The TAS subscale of the SSSC (Russo et al. 1993) comprised 12 questions. Again, participants endorse the choice that most describes what they like or feel, for example, “(a) I'd never do anything that's dangerous” or “(b) Sometimes I like to do things that are a little scary.” Responses that reflect TAS were assigned 1-point each, and were summed to create a score that ranged from 0 to 12 (higher scores indicate greater thrill and adventure-seeking tendencies). The measure demonstrates very good internal reliability (Cronbach's *α* = 0.82). The DAS subscale includes seven questions, each with a forced response option, for example, “(a) I would like to try marijuana” or “(b) I would never smoke marijuana.” Responses that reflect DAS are assigned 1-point each, and responses were summed to create a score that ranged from 0 to 7 (higher scores indicate more drug and alcohol seeking tendencies). The DAS subscale also demonstrates good internal reliability (Cronbach's *α* = 0.73).

### DNA collection

Participants' saliva samples were collected in Oragene vials (DNA Genotek, Ottawa, Ontario, Canada). DNA extraction was performed using a “Purifier” solution with alcohol precipitation per the manufacturer's protocol. The median yield of DNA from 2 mL of saliva captured in 2 mL of Oragene DNA was 110 *μ*g.

### SNP selection

Candidate genes were first identified from both published reviews (Kreek et al. 2004) and PubMed searches of human genetic studies using the following key words: sensation seeking, risk taking, gambling, smoking onset, and initiation. Multiple types of genetic studies were reviewed to assemble this list of candidate genes, including genome-wide association studies (GWAS), candidate gene studies, and family studies. In addition, four neural pathways were further examined in great detail: the serotonergic, dopaminergic, opioid, and cannabinoid pathways. This list of candidate genes was cross-referenced with the Gene Ontology Database (http://pid.nci.nih.gov/) and the Kegg Pathway database (http://www.genome.jp/) in order to confirm pathway information. Tagging SNPs for these genes were selected using data from the International HapMap Project (Release 21 with NCBI build 36; http://www.hapmap.org). SNPs were selected based on the following criteria: located either in the respective gene or no more than 10 kb upstream or downstream of the gene (to cover the regulatory regions); minor allele frequency (MAF) >5%, and not already represented by another tag SNP at a linkage disequilibrium (LD) of *r*^2^ > 0.80. For each candidate gene, all SNPs meeting selection criteria that were not in LD with another already chosen SNP (*r*^2^ ≤ 0.80) were also included, to ensure that candidate genes were adequately covered. Additionally, SNPs residing in coding regions (synonymous SNPs, nonsynonymous SNPs), regulatory regions (promoter, splicing site, 5-UTR, and 3-UTR), and noncoding regions were included. In addition, SNPs previously reported to be associated with smoking phenotypes (Bierut et al. 2007) were also included in the analyses. Table S1 enumerates the name, base pair position, and chromosome associated with each of the 565 SNPs examined in this study that were retained after the quality control, and also includes results of the logistic regression analyses conducted on each SNP separately, including best-fitted genetic model and parameter estimate for each SNP.

### Genotyping

DNA samples from a total of 1274 participants were sent for genotyping. Genotyping of the candidate SNPs was performed using an Illumina GoldenGate assay (Illumina, San Diego, CA). Ninety-three percent of the SNPs analyzed had Illumina SNP scores of >0.6. Genotyping of DNA samples (250 ng) was conducted following Illumina's standard 3-day protocol. The BeadArray reader (Illumina, Inc.) was used to autocall data from the SNP array. Cluster definitions for each SNP were determined using Illumina BeadStudio Genotyping Module (v. 2.3.41). SNP genotype assignments (calls) were made when a genotype yielded a quality value (Gencall score) of 95% or higher. Among the markers included in this study, only 1.5% had a Gencall score less than 95% (10 of 672). Seventy blind duplicate pairs were included, and the overall concordance of SNP genotype calls was greater than 99%.

### Statistical analyses

Univariable linear regression models were used to examine associations between social disinhibition score and all demographic and psychosocial covariates (age, gender, parental education, level of acculturation, TAS score, and DAS score). An overall multivariable model was then developed using these same demographic and psychosocial risk factors. In these regression analyses, gender and parental education were modeled as categorical variables, whereas age, acculturation, TAS scores, and DAS scores were all modeled as continuous covariates. However, in our descriptive data summary table, we also showed acculturation, TAS score, and DAS score results as categorical data (based on median splits) in addition to giving continuous data estimates such as mean, standard deviation, and range, in an effort to further describe results for these variables.

For each candidate SNP, allelic data were recoded into three potential genetic models: an additive, a dominant, and a recessive model. Three separate linear regression analyses were then conducted for each candidate SNP (one for each genetic model), controlling for age and gender.

Because we performed three regression analyses for hundreds of candidate SNPs, we used the Bayesian False Discovery Probability (BFDP) test to determine the chance of obtaining false-positive results due to the multiple comparisons (Wakefield 2007). We calculated BFDP values for the ten most statistically significant regression results for each risk model, using four levels of prior probability (0.01, 0.03, 0.05, and 0.07), a prior odds ratio (OR) of 1.5, and setting our threshold of BFDP noteworthiness at the recommended value of 0.8 (Wakefield 2007).

Principal components analysis was also conducted to test for possible underlying ethnic stratification, with the use of EIGENSTRAT software (Price et al. 2006). We first applied principal components analysis to the genotype data to infer continuous axes of genetic variation (eigenvectors). We then used the top axes of variation as covariates in a multiple regression analysis (described below).

The best-fitting genetic model (i.e., dominant, recessive, or additive) for each SNP with significant regression results as well as a BFDP value of <0.8 were examined simultaneously in a multiple linear regression model, which also included demographic and psychosocial risk factors and relevant principal component terms to adjust for underlying ethnic stratification. A final model of significant SNPs (adjusting for these other variables) was determined using a manual backwards elimination process (those SNPs with a *P*-value >0.05 in the multivariable model were removed).

## Results

DNA from 1274 enrolled youth was available for analysis. Of the individuals genotyped, 1132 had social disinhibition data available. However, due to additional missing data for parental education (*n* = 65), linguistic acculturation (*n* = 2), and on one of the SNPs of interest (rs4938056; *n* = 2), the final sample size available for the multivariable analyses was 1064.

Demographic characteristics and psychosocial risk factors of study participants are shown in Table 1. The cohort was evenly split by gender, the mean age of participants was 14.4 years (SD = 1.03), and overall, the mean social disinhibition score was very close to the middle of the range of values (3.3; SD = 1.9). Males had a significantly higher mean social disinhibition score than females (*F* = 34.4, df = 1, *P* ≤ 0.001). Age was also significantly associated with mean social disinhibition score (*F* = 13.7, df = 5, *P* < 0.001); youth ≥14 years of age had higher mean scores than youth 12 or 13 years of age.

**Table 1:** Demographic and psychosocial characteristics

	*N* (%)	Mean social disinhibition score (SD) (range: 1–7)	*P*-value
Overall	1064 (100.0)	3.3 (1.9)	
Gender			
Females (2)	538 (50.6)	3.0 (2.0)	<0.001
Males (1)	526 (49.4)	3.6 (1.8)	
Age (years)			
12	6 (0.6)	0.8 (0.8)	<0.001
13	234 (22.0)	2.6 (1.9)	
14	347 (32.6)	3.2 (2.0)	
15	319 (30.0)	3.7 (1.8)	
16	142 (13.3)	3.7 (1.7)	
17	16 (1.5)	3.6 (1.7)	
Mean (SD)	14.4 (1.03)		
Linguistic acculturation			
Low score (≤3)	317 (29.8)	2.7 (2.0)	<0.001
Medium score (>3 and <4)	379 (35.6)	3.4 (1.9)	
High score (≥4)	368 (34.6)	3.8 (1.8)	
Mean (SD)	3.5 (0.9)		
Range	1–5		
Parental education			
<High school	701 (65.9)	3.2 (1.9)	0.006
High school graduate	177 (16.6)	3.6 (1.9)	
>High school	186 (17.5)	3.5 (1.9)	
Thrill and adventure seeking (TAS)			
Low score (≤7)	565 (53.1)	2.5 (1.8)	<0.001
High score (≥8)	499 (46.9)	4.2 (1.7)	
Mean (SD)	6.8 (3.3)		
Range	1–12		
Drug and alcohol seeking (DAS)			
Low score (0)	537 (50.5)	2.4 (1.7)	<0.001
High score (≥1)	527 (49.5)	4.2 (1.7)	
Mean (SD)	1.2 (1.6)		
Range	1–7		

Youth with at least one parent who had completed high school or had attained additional education past high school reported higher mean social disinhibition scores compared with youth whose parents did not graduate high school (*F* = 5.2, df = 2, *P* = 0.006). The mean linguistic acculturation score for the cohort was 3.5 (SD = 0.9); participants with higher linguistic acculturation scores reported higher mean social disinhibition scores (*F* = 30.7, df = 2, *P* < 0.001) compared to their less acculturated counterparts. Participants' mean TAS and DAS scores were 6.9 (SD = 3.3) and 1.2 (SD = 1.6), respectively. Both scores were significantly and positively associated with mean social disinhibition score (*P* < 0.001 for both).

A total of 672 candidate SNPs were genotyped. Ten SNPs had a Gencall score of less than 95%, 78 additional SNPs failed the minor allele frequency test (MAF <0.05), and 19 SNPs failed the Hardy–Weinberg equilibrium test (*P* < 0.000001). A total of 565 SNPs were included in the analyses. There were 60 SNPs with *P* < 0.05 based on the best genetic model fit (additive, dominant, or recessive), adjusting for age and sex. After controlling for false discovery, we identified 11 SNPs with a statistically significant BFDP of <0.8 and a prior probability of 0.05.

For the principal components analysis, we used *N* = 1132 participants and 511 SNPs which were shown to be unassociated with the social disinhibition outcome in univariate analyses at a significance level of 0.05 based on the best genetic model fit (additive, dominant, or recessive model). We did not observe significant ethnic stratification in our data from the principal components analysis. Because only the top three eigenvalues (derived from the top three principal components) were significantly larger than the subsequent eigenvalues, we used these 3 largest principal components in our analyses (Tian et al., 2008; Nassir et al., 2009). We also considered controlling for the top 5 and top 10 largest principal components but found no significant difference in the association between SNPs and social disinhibition.

Six of the 11 SNPs were found to be significant at *α* = 0.05 level in the final multivariable model, which included demographic and psychosocial characteristics as well as the three largest principal components terms. Two of these SNPs are in the serotonin pathway (rs9534511 on *HTR2A* and rs4938056 on *HTR3B*), and one is in an intronic region of an opioid receptor gene (rs1998220 on *OPRM1*). One SNP (rs1003921) resides in an intronic region of *KCNC1*, one SNP (rs16116) is in an intergenic region downstream of *NPY*, and one SNP (rs16870286) is part of *LINC00518*, a miscellaneous RNA gene that codes for an uncharacterized protein. Information about these SNPs, including model used, multivariable *P*-value, and allelic frequency, is shown in Table 2.

**Table 2:** Distribution of significant genes/SNPs that remained in final multivariable model, after controlling for false discovery by new experimenter status

Genes (SNP)	Chromosome	Genetic model	Minor allele	*N* (%) 1064 (100.0)	*P*-value	MATCh minor allele %
*OPRM1* (rs1998220)						
AA	6	A	G	408 (38.4)	0.003	38.3
AG				496 (46.6)		
GG				160 (15.0)		
*HTR2A* (rs9534511)						
AA	13	D	G	384 (36.1)	0.001	40.1
AG/GG				680 (63.9)		
*HTR3B* (rs4938056)						
AA	11	D	G	440 (41.4)	0.01	34.8
AG/GG				624 (58.6)		
*NPY* (rs16116)						
GG	7	D	A	283 (26.6)	0.004	47.9
AG/AA				781 (73.4)		
*LINC00518* (rs16870286)						
GG	6	R	A	465 (43.7)	0.029	34.4
AG/AA				599 (56.3)		
*KCNC1* (rs1003921)						
AA	11	R	G	775 (72.8)	0.011	15.2
AG/GG				289 (27.2)		

A, additive; D, dominant; R, recessive.

Parameter estimates for the final multivariable model are shown in Table 3. Age, linguistic acculturation, TAS, and DAS were all significantly positively associated with increased risk of social disinhibition (*P* < 0.001). Each 1-year increase in age was estimated to increase social disinhibition score by 0.20 points (95% CI = 0.12–0.29), and each 1-point increase in a participant's overall linguistic acculturation score was expected to increase their social disinhibition score by 0.30 points (95% CI = 0.20–0.41). Similarly, each 1-unit increase in TAS or DAS score is expected to increase participants' social disinhibition score by 0.20 (95% CI = 0.17–0.23) and 0.45 points (95% CI = 0.40–0.51), respectively. Gender and parental education, however, were not significantly associated with social disinhibition score (*P* = 0.54 and *P* = 0.13, respectively).

**Table 3:** Multiple linear regression results for multivariable model for social disinhibition, including demographic/psychosocial, SNP, and principal components data as independent variables in model (*N* = 1064)

	Estimate	95% CI	*P*-value
Social and psychological factors			
Age	0.20	0.12, 0.29	<0.001
Female	0.06	−0.13, 0.24	0.544
Parental education			0.125
<HS	0.0 (referent)	–	–
HS grad	0.20	−0.04, 0.45	0.098
>HS	−0.10	−0.33, 0.15	0.449
Acculturation	0.30	0.20, 0.41	<0.001
TAS score	0.20	0.17, 0.23	<0.001
DAS score	0.45	0.40, 0.51	<0.001
Gene (SNP)			
*OPRM1* (rs1998220)	0.19	0.06, 0.32	0.003
*HTR2A* (rs9534511)	0.32	0.14, 0.50	<0.001
*HTR3B* (rs4938056)	0.23	0.06, 0.41	0.010
*NPY* (rs16116)	−0.29	−0.48, −0.09	0.004
*LINC00518* (rs16870286)	−0.30	−0.56, −0.03	0.029
*KCNC1* (rs1003921)	−0.64	−1.13, −0.15	0.011

Controlled for top three principal components factors.

Minor alleles for three of the SNPs were associated with increased risk of social disinhibition (rs1998220, rs9534511, and rs4938056), while the other three SNPs' minor alleles were associated with protective effects (rs16116, rs16870286, and rs1003921). For those SNPs that were positively associated with increased social disinhibition score, parameter estimates (the amount a minor allele was estimated to change social disinhibition score by) ranged from 0.19 (rs1998220; 95% CI = 0.06–0.32, *P* = 0.003) to 0.32 (rs9534511; 95% CI = 0.14–0.50, *P* < 0.001). Parameter estimates for SNPs that were negatively associated with increased social disinhibition score ranged from −0.29 (rs16116; 95% CI = −0.48 to −0.09, *P* = 0.004) to −0.64 (rs1003921; 95% CI = −1.13 to −0.15, *P* = 0.011).

## Discussion

We identified six SNPs that were significantly associated with social disinhibition among Mexican-American youth. These included two SNPs in the serotonin pathway, one in an intronic region of an opioid receptor gene, one in an intronic region of a potassium channel gene, one in an intergenic region near the neuropeptide Y gene, and one in a gene that codes for an uncharacterized RNA protein.

The serotonin pathway plays a role in neuropsychiatric conditions such as depression, anxiety, and autism, and also plays a role in regulating social behavior (Oreland et al. 2010; Kinast et al. 2013). As Zuckerman (1994) observed, serotonin appears to act as a behavioral inhibitor. We observed two variants in the serotonin pathway (in *HTR2A* and *HTR3B*) that were significantly associated with an increased risk of social disinhibition; our findings appear consistent with current knowledge about the serotonin pathway. Wilkinson et al. (2012) also reported an association between a SNP near *HTR2A* (9567732) and cigarette experimentation among adolescents, as well as an association between another gene in the serotonin pathway (*HTR1B*) and cigarette experimentation. Since cigarette experimentation is associated with increased social disinhibition, our study results are also consistent with these findings.

The *OPRM1* gene encodes the mu-opioid receptor, which has been associated with drug addiction, including nicotine dependence (Zhang et al. 2006). The *OPRM1* SNP that was significantly associated with an increased risk of social disinhibition in our study (rs1998220) was part of a 3-SNP haplotype found in a previous study to be associated with increased feelings of energy, euphoria, and stimulation, and was also independently associated with amphetamine response (Dlugos et al. 2011). We previously reported that another SNP (rs9567732) on *OPRM1* was associated with adolescent cigarette experimentation among committed never smokers (Wilkinson et al. 2012); the SNP identified in this study (rs1998220) is not in LD with rs9567732. These findings are consistent with our results, because drug dependence and cigarette experimentation are associated with social disinhibition (McGue et al. 2006; Wilkinson et al. 2011), as is increased sensitivity to sensations of energy and euphoria (Stoops et al. 2007).

*KCNC1* is a gene that codes for a potassium voltage-gated channel protein. More specifically, this gene encodes a membrane protein which mediates potassium-ion permeability of membranes. This channel is often expressed in neurons, and it enables them to repetitively fire at a high frequency (Rudy and McBain 2001; Pedroarena 2011). In mouse models, mice that lacked two potassium voltage-gated channels (Kv3.1 encoded by *KCNC1* as well as Kv3.3) exhibited tremor, ataxia, and alcohol hypersensitivity (McMahon et al. 2004; Joho and Hurlock 2009), as well as altered circadian rhythms (Kudo et al. 2011).

*NPY* encodes neuropeptide Y, which is commonly expressed throughout the central and peripheral nervous systems. *NPY* expression helps to inhibit anxiety, and genetic variations in the expression of *NPY* modulate emotion and stress response (Zhou et al. 2008; Benarroch 2009). A study by Zhou et al. (2008) found that several rare 7-SNP haplotypes were associated with higher expression of *NPY* and thus with lower trait anxiety. Similarly, we observed that the minor allele of rs16116, downstream of *NPY*, had a protective effect upon social disinhibition score (was associated with decreased social disinhibition). *NPY* is also associated with neuropsychiatric disorders such as anxiety, depression, and bipolar disorder (Coccaro et al. 2012). A genetic variant in *NPY* (SNP rs16147) has also been associated with an increased risk of smoking tobacco (Mutschler et al. 2012); however, this SNP is not in LD with rs16116.

The *LINC00518* gene on chromosome 6 codes for an uncharacterized RNA protein, and little is known about the function of this protein. However, chromosomal duplications and deletions in or near this gene seem to be linked to several behavioral disorders, such as ADHD, autism-spectrum disorders, and developmental delays (Lionel et al. 2011; Celestino-Soper et al. 2012). This gene is also very close (8126 base pairs away) to the *TFAP2A* gene (http://www.genecards.org), which encodes transcription factor AP-2 *α*. Transcription factor AP-2 helps to regulate neural development and neural gene expression (Oreland et al. 2002).

All demographic and psychosocial risk factors analyzed in this study (age, gender, linguistic acculturation, parental education, and TAS and DAA scores) were significantly associated with social disinhibition score in univariable analyses (Table 1). However, in the multivariable model (Table 3), gender and parental education were no longer significantly associated with SD score, after adjusting for all other variables in the model. Steinberg et al. (2008) observed that among adolescents, sensation seeking increased between 10 and 15 years of age, and then either remained stable or decreased thereafter. Our social disinhibition univariable results showed a very similar pattern. In our univariable results, males tended to have higher social disinhibition scores than females. Other studies have also reported higher disinhibition as well as higher total sensation-seeking scores for males (Zuckerman et al. 1991; Zuckerman 1994; Roberti 2004).

In this study, acculturation was significantly associated with social disinhibition, both in univariable and multivariable analyses (*P* < 0.001). Consistent with previous findings (Wilkinson et al. 2012), youth with higher acculturation levels tended to have higher social disinhibition scores. Although in this study, we have focused on social disinhibition as being problematic, it is possible that in immigrants, this trait might also be adaptive. The desire to immigrate has been associated with increased sensation-seeking tendencies (Winchie and Carment 1988); therefore, social disinhibition might be associated with characteristics such as increased venturesomeness, a greater likelihood of staying in school, or of being involved in the larger community. However, it is possible that this advantage is greater in parents (the immigrants themselves) than in offspring.

Parental education was significantly associated with social disinhibition in a univariable model (*P* = 0.006), but not in the final multivariable model (*P* = 0.125). Youth with higher parental education levels (high school or greater) tended to have higher social disinhibition scores than youth whose parents did not finish high school. Parental education was likely excluded from the final multivariable model because linguistic acculturation and parental education were weakly (*r* = 0.23) but significantly correlated (*P* < 0.001), and the univariable *F* statistic was lower for education than acculturation. Likewise, gender might also have been excluded from the multivariable model because it was significantly correlated with TAS score (*r* = −0.36, *P* < 0.001), and had a smaller univariable *F* score than did TAS score.

Sensation-seeking tendencies, similar to other behavioral traits such as depressive symptoms, anger traits, and sensitivity to stress (Mizuno et al. 2006; Baud et al. 2007; Shifman et al. 2008; Guo and Tillman 2009), vary by gender (Russo et al. 1993; Wilkinson et al. 2011, 2012). Because gender differences are seen across all aspects of sensation seeking, and because social disinhibition score differed significantly by gender in our univariable analysis (Table 1), we completed an exploratory stratified analysis by gender. One SNP demonstrated a significant SNP by gender interaction (rs16870286 on *LINC00518*; Wald *χ*^2^(1 df) = 4.26; *P* = 0.039). Among the remaining SNPs, while different SNPs were significantly associated with social disinhibition for males than for females, this was likely due to reduced sample sizes when stratifying by gender, as the magnitude of the effect of the SNPs differed between males and females but the direction of each SNP's association was the same.

Strengths of this study included that the data used were from a population-based cohort, and had fairly equal numbers of boys and girls. Validated measures were used for linguistic acculturation and all three sensation-seeking subscale (social disinhibition, TAS, and DAS) scores. Psychosocial and demographic data were obtained from the participants directly, instead of collecting these data from a parent or guardian proxy. The use of personal digital assistants to collect participant information also ensured a high level of participant privacy as well as valid and high-quality data. Finally, this study focused on an understudied population—that of low-income Mexican-American youth.

The primary limitation of this study is the lack of a replication sample with which to validate our findings; therefore, our study results should only be viewed as preliminary. However, the absence of an independent replication sample is fairly typical in studies of minority populations. Second, all participants in this study were Mexican-origin youth, and thus, results might not generalize to youth of other races, ethnicities, or countries of origin. Social disinhibition data, as well as other psychosocial data, were self-reported, and might have been subject to recall bias. Although we tried to include all known candidate genes for sensation-seeking tendencies and cigarette smoking behaviors in our analysis, our list was not exhaustive. For example, SNPs on the *CTNNA2* gene, which has been associated with excitement seeking in GWAS and a meta-analysis (Terracciano et al. 2011), were not included in this study. Potential candidate SNPs reported by McGue et al. (2013) in a recent GWAS of behavioral disinhibition were also not included. Similarly, other candidate genes may not have been included in this analysis either because they were missed or have been recently identified. Finally, this was a cross-sectional study, and we cannot determine causality. This also means that significant SNPs (or the genes that they are associated with) in the model might not be functionally significant.

In conclusion, this study presents six SNPs that were significantly associated with social disinhibition in Mexican-American youth. These results add to our knowledge of genetic risk factors for social disinhibition, an aspect of sensation seeking that has been strongly associated with cigarette and alcohol experimentation among adolescents. Additional research is needed to verify whether these SNPs are associated with social disinhibition among youth of different ethnicities and nationalities, and to elucidate whether/how these SNPs functionally contribute to social disinhibition and associated behaviors.

## Supporting Information

**Table S1**. Enumerates the name, base pair position, and chromosome associated with each of the 565 SNPs examined in this study that were retained after the quality control, and also includes results of the logistic regression analyses conducted on each SNP separately, including best-fitted genetic model and parameter estimate for each SNP.
